# Fish oil diet may reduce inflammatory levels in the liver of middle-aged rats

**DOI:** 10.1038/s41598-017-06506-3

**Published:** 2017-07-24

**Authors:** Yingqiu Li, Fan Zhao, Qiayu Wu, Mengjie Li, Yingying Zhu, Shangxin Song, Jing Zhu, Yafang Ma, He Li, Xuebin Shi, Xinglian Xu, Guanghong Zhou, Chunbao Li

**Affiliations:** 1 Key Laboratory of Meat Processing and Quality Control, MOE, Key Laboratory of Meat Processing, MOA, Jiangsu Innovation Center of Meat Production and Processing, Nanjing Agricultural University, Nanjing, 210095 P.R. China; 2 Guangxi Vocational College of Technology and Business, Nanning, 530008 Guangxi P.R. China; 3 School of Food Science, Nanjing Xiaozhuang University, Nanjing, 211171 P.R. China

## Abstract

The impact of dietary soybean oil, lard and fish oil on physiological responses in middle age is little studied. In this study, we investigated the changes of oxidative stress, inflammatory cytokines, telomere length, and age-related gene expression in the liver of middle-aged rats in response to the above three fat diets. Male *Sprague Dawley* rats (12 months old) were fed AIN-93M diets for 3 months, in which soybean oil was equivalently replaced by lard or fish oil. As compared to the lard diet, intake of fish oil diet significantly decreased body weight gain, white blood cell count, and levels of hepatic triacylglycerol, total cholesterol, fat accumulation, low-density lipoprotein, oxidative stress and inflammatory cytokines (*P < *0.05), but increased telomere length (*P < *0.05). On the other hand, lard diet and soybean oil diet showed great similarity in the above variables. PCR array analysis further indicated that fish oil diet significantly down-regulated gene expression related to inflammatory response, apoptosis, DNA binding, proteostasis and telomere attrition. Differentially expressed genes were enriched in the complement and coagulation cascades pathways. Such physiological and molecular responses could be due to different fatty acid composition in fish oil, lard and soybean oil.

## Introduction

Fat is an important component of human diet and its nutritional value is largely dependent on the fatty acid composition^1^. Different sources of fat have different applications. Lard was mainly applied to bakery and food homemaking in western countries many years ago and it has been replaced by plant oils, including soybean, sunflower and olive oils in terms of health concerns^2, 3^. However, lard is still widely consumed in some developing countries. Some people even believe that lard is not bad as we image^4^. Fish oil is usually applied as a supplement agent because it contains high levels of n-3 fatty acids and can prevent some diseases^5^.

Fatty acid composition in fat diets has been shown associated with oxidation and inflammation. Saturated fatty acids (SFAs) are more resistant to oxidation because they do not contain unsaturated bonds. However, high-saturated-fat diets may increase the risk to inflammation level in murine models^6^. On the other hand, n-3 polyunsaturated fatty acids (PUFAs) have anti-oxidative activity because NF-κB pathway can be inhibited by eicosahexaenoic acid (EPA), docosahexaenoic acid (DHA) and their metabolites^7^. The deficiency of n-3 PUFAs may promote lipogenic gene expression and hepatic steatosis through the liver X receptor^8^. On the contrary, n-6 PUFA-rich diets may lead to higher levels of lipid peroxidation and DNA oxidative breaks in rat tissues and blood during aging, while MUFA-based diets showed less oxidation^9^. The n-3 PUFAs are thought to systemically decrease inflammatory responses by down-regulating inflammatory gene expression via NF-κB inactivation^10^. However, n-6 fatty acids, in particular to linoleic acid (18:2 n-6), may exert a pro-inflammatory effect^11^. Monounsaturated fatty acids (MUFAs), including oleic acid (OA), have been shown to play a dual role in inflammation^6, 12–14^.

Oxidative stress has been recognized as a critical contributor to many physiological changes, in particular to the aging process^15, 16^, which is characterized as a chronic and subclinical inflammatory state^17^. The aging process is normally accompanied with genomic instability, telomere attrition, epigenetic alterations, loss of proteostasis, mitochondrial dysfunction and cellular senescence^18^. Middle age is an earlier stage of the aging process, during which gradual physical changes and some chronic illness may occur^19^. Such changes would affect the outcomes at old ages^20^. Many studies have emphasized the impact of diets on aging. However, few data are available on the impact of dietary fats on physiological responses at middle ages.

To this end, we investigated how intake of soybean oil, lard and fish oil affected oxidative and inflammatory status, age-related gene expression, telomere length and other related parameters of middle-aged rats. The underlying mechanism was also proposed.

## Results

### Fat accumulation

**Figure 1:**
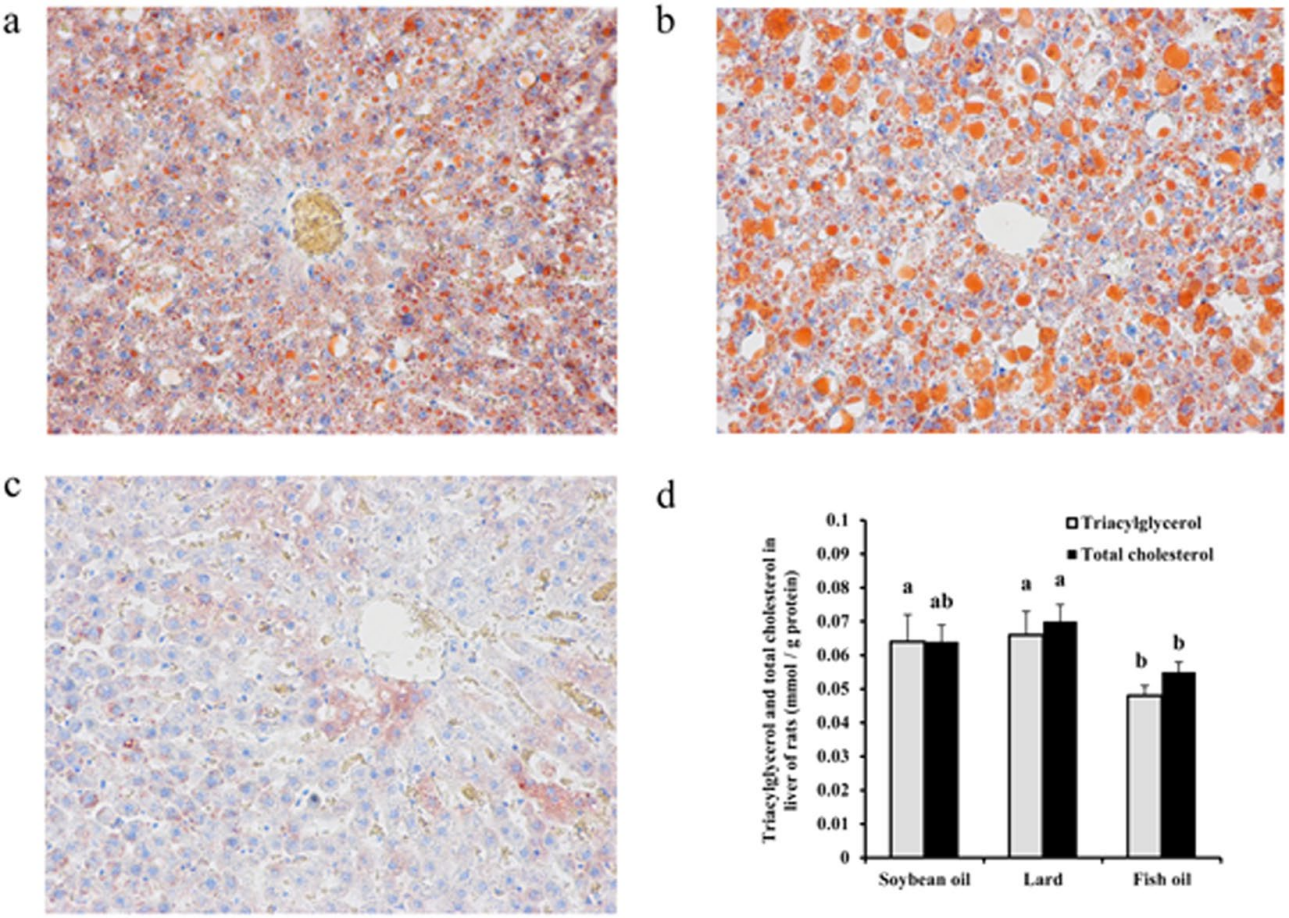
Hepatic fat accumulation of rats fed with soybean oil, lard and fish oil diets. (**a**) Hepatic section stained with Oil Red O from the soybean oil group. (**b**) Hepatic section stained with Oil Red O from the lard group. (**c**) Hepatic section stained with Oil Red O from the fish oil group. Adipocytes were stained red and nuclei were stained blue. (Magnification ×200). (**d**) Hepatic triacylglycerol and total cholesterol. Values are shown as means ± SE (n = 11). Different letters indicate significant difference (*P* < 0.05).

Histological observations with Oil Red O staining indicated that the quantity and size of hepatic fat droplets in the lard group were greater than those in the soybean oil and fish oil groups (Fig. 1a–c). Correspondingly, hepatic triacylglycerol (TAG) and total cholesterol (TC) in the lard group were significantly higher than those in the fish oil group (Fig. 1d, *P* < 0.05). No significant difference existed between the lard group and the soybean oil group (Fig. 1d, *P* > 0.05). The lard group also showed greater body weight gain and liver index than the fish oil and soybean oil groups (*P* < 0.05, Table 1), although the actual body weight and feed intake were not significantly different among the three diet groups (*P* > 0.05, Table 1). In blood, TAG, TC and low-density lipoprotein (LDL) were higher in the lard group than those in the fish oil and soybean oil groups (*P* < 0.05, Table 2). Insulin resistance index was not significantly different among the three diet groups (*P* > 0.05).

**Table 1:** Food intake, body weight gain and indices of rats after feeding for 3 months (means ± standard errors, n = 11).

Items	Soybean oil	Lard	Fish oil
Average daily feed intake (g)	21.74 ± 0.96^a^	23.31 ± 0.81^a^	24.12 ± 0.64^a^
Initial body weight (g)	784.40 ± 13.35^a^	800.60 ± 11.49^a^	816.20 ± 16.29^a^
Final body weight (g)	881.10 ± 17.49^a^	933.20 ± 21.10^a^	900.00 ± 16.79^a^
Body weight gain (g)	96.70 ± 6.98^b^	132.60 ± 11.73^a^	83.80 ± 7.16^b^
Liver index (mg/g)	24.42 ± 0.39^b^	26.17 ± 0.58^a^	23.58 ± 0.57^b^

**Table 2:** Blood parameters of rats fed with soybean oil, lard or fish oil diets (means ± standard errors, n = 11).

Items	Soybean oil	Lard	Fish oil
Glu (mmol/L)	5.27 ± 0.20^a^	5.47 ± 0.27^a^	4.89 ± 0.18^a^
TAG (mmol/L)	1.59 ± 0.11^b^	1.99 ± 0.14^a^	1.09 ± 0.13^c^
TC (mmol/L)	3.06 ± 0.12^b^	3.50 ± 0.17^a^	2.76 ± 0.11^b^
HDL (mmol/L)	1.20 ± 0.06^a^	1.02 ± 0.04^b^	1.00 ± 0.05^b^
LDL (mmol/L)	1.68 ± 0.06^b^	1.94 ± 0.09^a^	1.48 ± 0.06^b^
White blood cell count (WBC, 10^9^/L)	8.88 ± 0.51^b^	10.68 ± 0.70^a^	8.20 ± 0.28^b^
Insulin resistance index (IRI)	13.96 ± 2.47^a^	15.16 ± 1.55^a^	12.29 ± 1.09^a^

### Oxidative status

**Figure 2:**
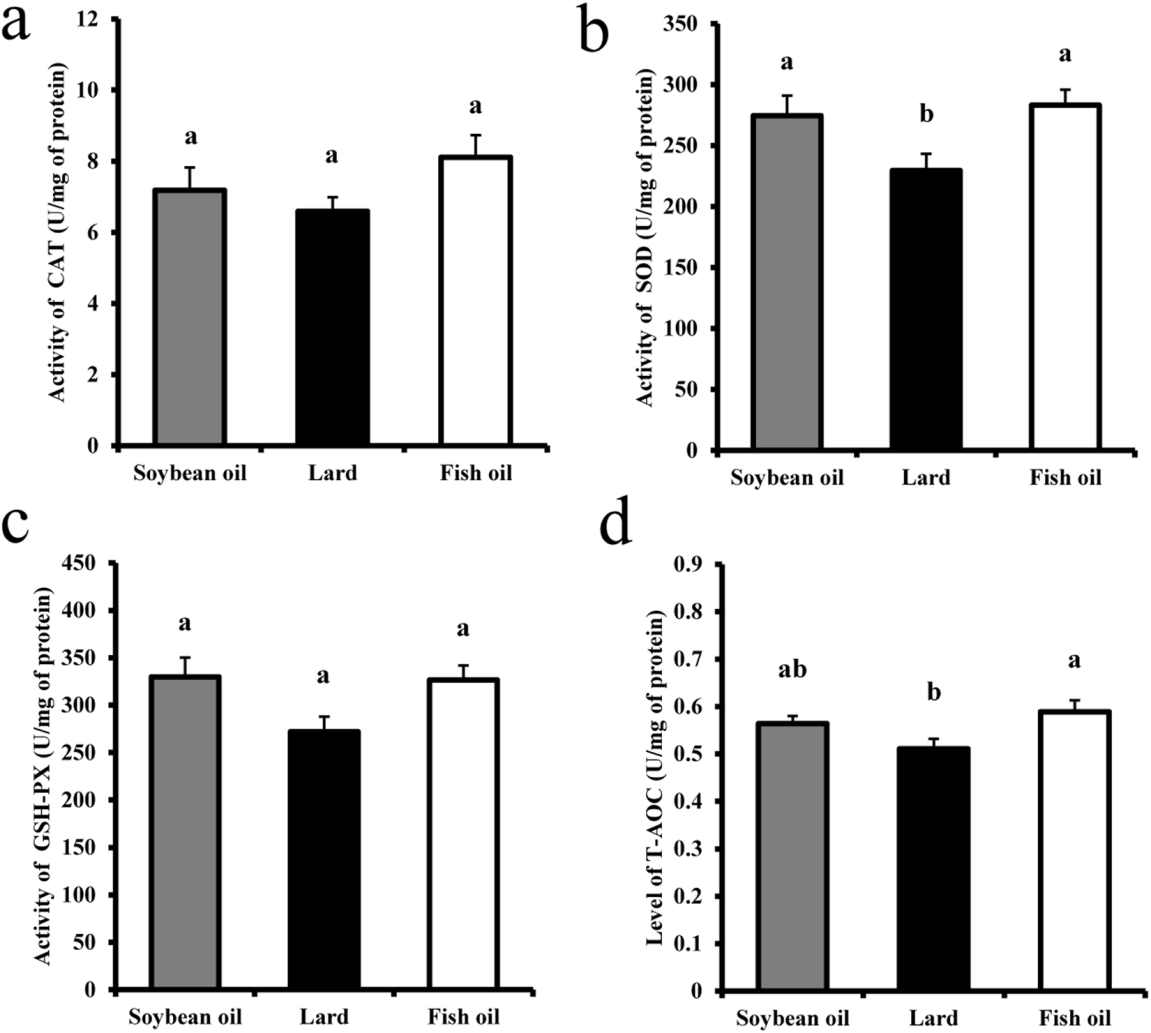
Antioxidant enzyme activities level of rats fed with soybean oil, lard and fish oil diets. (**a**) Catalase activity; (**b**) Superoxide dismutase activity; (**c**) Glutathione peroxidase activity; (**d**) Total antioxidant capacity. Values are shown as means ± SE (n = 11). Different letters indicate significant difference (*P* < 0.05).

Catalase (CAT), superoxide dismutase (SOD), glutathione peroxidase (GSH-PX), and total antioxidant capacity (T-AOC) were analyzed to indirectly evaluate reactive oxidative species (ROS) production in the liver (Fig. 2). The activities of SOD and T-AOC in the fish oil group were higher than those of the lard group (*P* < 0.05, Fig. 2b and d). Although a strict Student-Newman-Keuls test indicated no significant difference in CAT and GSH-Px activities among three diet groups (*P* *>*0.05, Fig. 2a and c), the moderately strict Duncan’s multiple comparisons showed a significant difference in CAT and GSH-Px activities between fish oil and lard groups (Figure S1). The soybean oil group did not exhibit any significant difference in these variables from the fish oil group (*P* > 0.05, Fig. 2). This indicates that intake of lard may impair the antioxidant activity to a greater extent than fish oil.

### Inflammatory status

**Figure 3:**
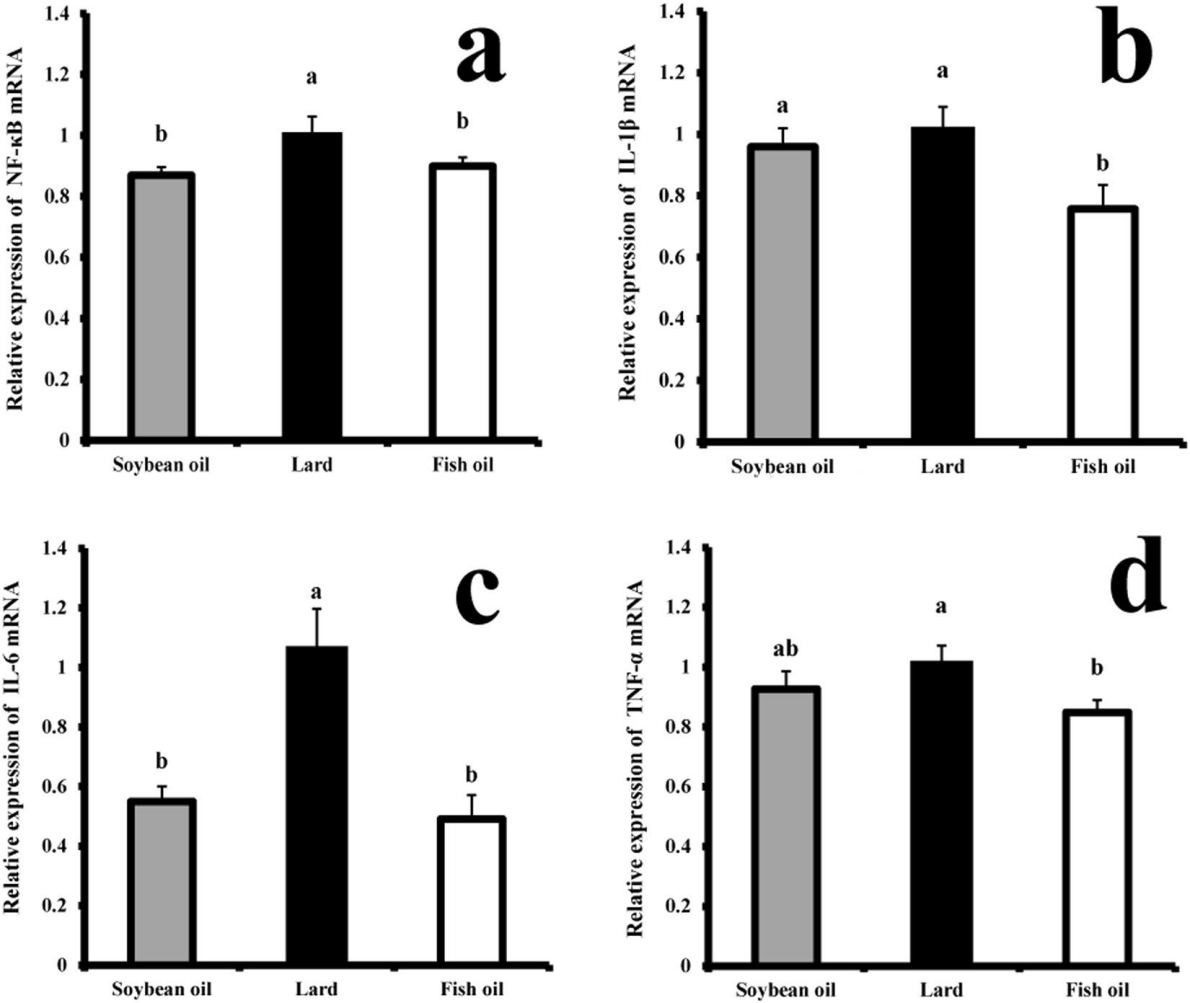
Hepatic mRNA levels of inflammatory cytokines of rats fed with soybean oil, lard and fish oil diets. (**a**) NF-κB; (**b**) IL-1β; (**c**) IL-6; (**d**) TNF-α. The mRNA levels were determined by RT-PCR. Different letters indicate significant difference (*P* < 0.05).

White blood cell count (WBC) in the soybean oil and fish oil groups were significantly lower than the lard group (*P* < 0.05, Table 2). The RT-PCR results indicated that mRNA levels of NF-κB, IL-1β, IL-6 and TNF-α were lower in the fish oil group than those in the lard group (*P* < 0.05, Fig. 3). No significant difference was observed in NF-κB and IL-6 mRNA levels between the soybean oil group and the fish oil group (*P* > 0.05, Fig. 3a and c). The IL-1β and TNF-α mRNA levels did not differ between the soybean oil group and the lard group (*P* > 0.05, Fig. 3b and d).

### Aging-related gene expression

**Figure 4:**
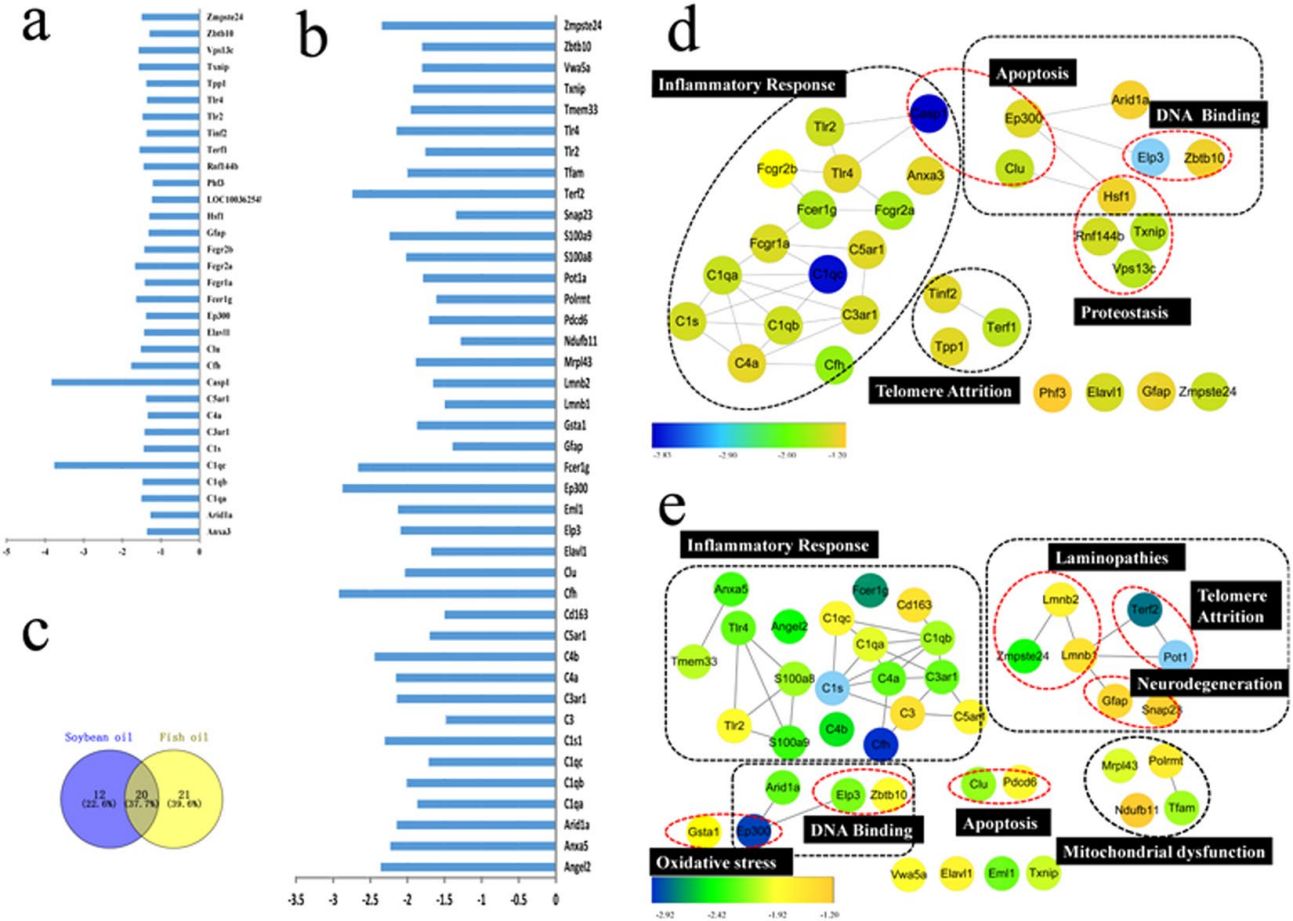
Aging-related PCR array of rat livers in response to soybean oil and fish oil as compared to the lard group. (**a**) 32 differentially expressed genes in the soybean oil group as compared to the lard group (P < 0.05); (**b**) 41 differentially expressed genes in the fish oil group as compared to the lard group (P < 0.05); (**c**) Venn plot of differentially expressed genes; (**d**) Gene set network of hepatic aging PCR array analysis of the soybean oil group as compared to the lard group. (**e**) Gene set network of hepatic aging PCR array analysis of the fish oil group as compared to the lard group. The networks were generated by STRING10 (http://string-db.org/) and Cytoscape 3.3.0. The node color change from blue to orange represents an increase of P value from −2.83 to −1.20 in the soybean oil group, and −2.92 to −1.20 in the fish oil group.

As shown above, intake of lard induced the middle-aged rats to substantially different physiological responses from the other two fat diets. To further explore the underlying molecular mechanisms, aging-related PCR array analyses were performed and mRNA data were compared in which the lard group was set as control. The results revealed that 41 and 32 genes were down-regulated in the fish oil group and the soybean oil group, respectively, as compared to the lard group (*P* < 0.05, Fig. 4a and b). Of these genes, caspase 1 and c1qc showed the greatest changes in the soybean oil group (actual change folds were −3.83 and −3.76, respectively, Fig. 4a). In the fish oil group, the absolute change folds of 20 genes were greater than 2.0 (Fig. 4b). The Venn plot further indicated that 12 and 21 differentially expressed genes in the soybean oil group and the fish oil group were specific for the diets, respectively (Fig. 4c). In addition, forkhead box O 1 (*FOXO1*) was down-regulated by 1.35 folds in the soybean oil group (*P* >0.05) and 2.13 folds in the fish oil group(P < 0.05).

Gene set analysis of PCR array data demonstrated that 16, 3, 2, 4 and3 of 32 differentially expressed genes in the soybean oil group genes were related to inflammatory responses, apoptosis, DNA binding, proteostasis and telomere attrition, respectively (Fig. 4d). The gene set enrichment analysis further indicated that those genes were involved in the pathways of complement and coagulation cascades, oxidative damage, complement activation and toll-like receptor signaling (Supplementary Table S1). Of 41 differentially expressed genes in the fish oil group, 19, 2, 2, 3, 2, 2 and 4 genes were associated with inflammatory responses, apoptosis, DNA binding, telomere attrition, laminopathies, neurodegeneration, oxidative stress and mitochondrial dysfunction respectively (Fig. 4e). The enrichment analysis matched these genes with six pathways, i.e., complement and coagulation cascades, oxidative damage, complement activation, toll like receptor signaling, mitochondrial gene expression, and FAS pathway and stress induction of HSP regulation (Supplementary Table S2).

### Telomere length

**Figure 5:**
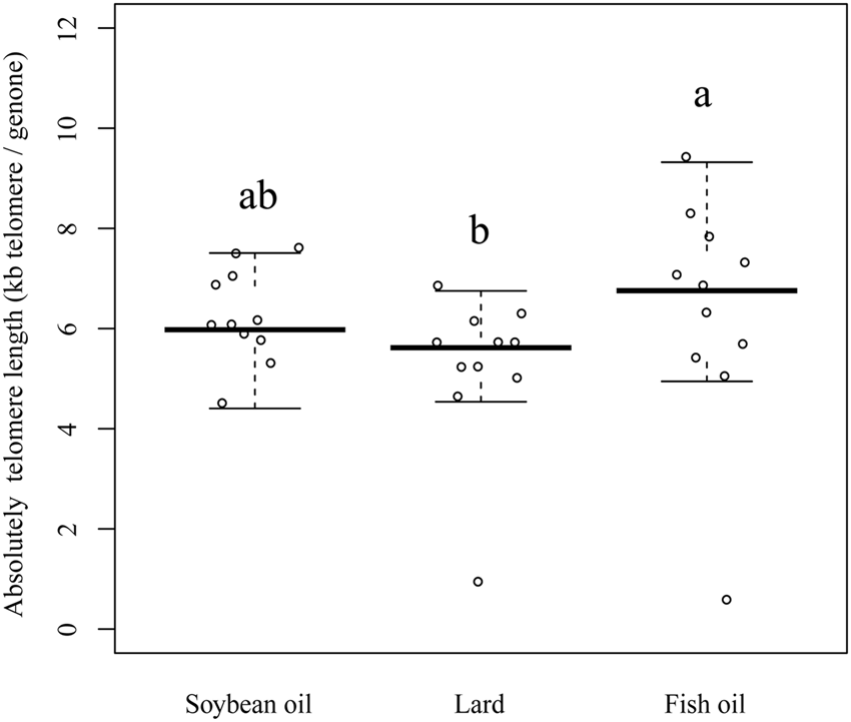
Hepatic absolute telomere length of rats fed soybean oil, lard and fish oil diets. Values are shown as means ± SE (n = 11). Different letters indicate significant difference (*P* < 0.05).

Telomere length was considered as a good indicator for the aging process^18^. The hepatic absolute telomere length (aTL) was measured by RT-PCR. The results indicated that the aTL values were significantly greater (*P* < 0.05, Fig. 5) for the fish oil group than those of the lard group, but no difference was observed for the soybean oil group from any of the other two groups (*P* > 0.05). Thus, intake of fish oil may retard telomere attrition for the middle-aged rats as compared to the intake of lard.

## Discussion

Increasing evidence indicates that the type of dietary fatty acids has a certain effect on animal health by altering lipid metabolism^21–23^. For example, intake of stearic acid leads to dramatically reduced visceral fat^24^, even if stearic acid can be more easily stored in liver at intermediate postprandial time points (24~48 h) than linolenic or oleic acid^25^. Palmitoleic acid and conjugated linoleic acid may prevent fat deposition in the liver^26, 27^. Furthermore, intake of DHA and arachidonic acid (ARA) can significantly reduce hepatic weight, serum TAG and TC, epididymal fat as compared to palmitic acid, which would be associated with enhanced fatty acid β-oxidation, down-regulated mRNA fatty acid synthase and sterol regulatory element binding protein-1c expression^28^. And thus, intake of long-chain saturated fatty acids (the number of carbon skeletons greater than 12) may increase lipid deposition, while monounsaturated or polyunsaturated fatty acids would inhibit lipid deposition. Such an effect could be dependent upon the age of studied subjects and the type of fatty acids.

Age effect is relatively complex, because the level of inflammation varies greatly with age during which Toll-like receptors (TLRs) play a critical role in the induction of inflammatory and immune responses^29^. In young rats, SFAs may have a greater contribution to pro-inflammatory processes than other fatty acids, because SFAs can be served as ligands for TLR-2 and TLR-4, resulting in induction of proinflammatory gene transcription via activation of NF-κB signaling cascades^30^. The n-6 fatty acids (e.g., linoleic acid), have been shown to exert proinflammatory effect^31^ and it could be converted into ARA. In young TLR4 knockout mice, EPA and DHA were found to inhibit the TLR-4 signaling pathway. However, n-3 PUFA supplementation (1.68 g EPA and 0.72 g DHA/d) in old subjects for 3 month showed greater potential to decrease inflammatory cytokines (IL-1β, IL-6, TNF-α) than for young ones^32^. The n-3 PUFA may alleviate age-related diseases by decreasing the level of inflammation^33^. The above results focused on the diet or age effects in different models, including high–fat-diet model. The diet formulations were quite different from the normal diet.

Therefore, the present study was intend to reveal how fatty acids in diet affect fat accumulation, oxidative status, inflammatory status, aging-relate gene expression and telomere length in the middle-aged rats.

Soybean oil, lard and fish oil showed significantly different fatty acid composition. Soybean oil is characterized by high levels of n-6 PUFAs (53.67%) and fish oil contains high levels of n-3 PUFAs (15.79% in EPA and 12.28% in DHA), while lard is largely composed of SFAs and MUFAs (43.59% and 39.99% respectively, Supplementary Table S3). The different fatty acid composition in diets may have associations with hepatic fat accumulation as well as body weight gain. Histological observations indicated a relatively moderate hepatic fat accumulation in the soybean oil group compared to lard and fish oil group. OA was the most abundant MUFAs in diet and blood^34^. OA can increase fat accumulation in hepatocytes by combining more specifically with acyl-CoA to form TAG than SFAs^34^. In the present study, the OA contents were 23.44%, 37.81% and 17.78% in soybean oil, lard and fish oil, respectively (Table S3). This may partially account for the highest hepatic fat accumulation and body weight gain in the lard group. The fish oil was more competent to reduce fat accumulation and body weight gain than soybean oil and lard just because of higher levels of n-3 fatty acids. Similar findings were observed that supplementation of 5% n-3 PUFA (EPA + DHA) in diet resulted in a complementary decrease in total body weight gain in young rats^35^. The n-3 PUFAs can not only increase insulin sensitivity, but also enhance lipid oxidation under the regulation of PPAR-α^7^. Long-chain n-3 PUFAs, e.g. EPA and DHA in fish oil may decrease the levels of plasma triglycerides^36, 37^. In this regard, it seems better for fish oil to prevent fat accumulation as compared to lard and soybean oil. This is because the deficiency of n-3 PUFAs may increase gene expression involved in lipogenesis under the regulation of liver X receptors^8^. The highest liver index and body weight gain in the lard group could be attributed to those of hepatic fat accumulation. In addition, higher SFAs in lard could cause fat accumulation. The SFAs, e.g. palmitic acid, may inhibit autophagy by inducing caspase-dependent Beclin 1 cleavage, and speed up the process of apoptosis^38^. Inhibition of autophagy in cultured hepatocytes and mouse liver might increase lipid storage^39^. Reduced autophagy in the liver may contribute to hepatic fat accumulation and further increase the incidence of metabolic syndromes in aged subjects^40^. The underlying mechanism may be that autophagy can not only decrease TAGs and lipid formation, but also increase TAG breakdown because TAGs and lipid droplet structural proteins co-localize in autophagic compartments^39^. And thus autophagy may protect against fatty acid-induced lipotoxicity, including aging and metabolic disorders^38, 41^. Autophagy also preserved a subset of old haematopoietic stem cells from replication stress by preventing entry into an activated state, which may improve old haematopoietic stem cell function therefore improve health and longevity^42^. The associations between autophagy and intake of soybean oil, lard and fish oil need further studies. Transmission electron microscopy and quantification of Atg8/LC3 and Atg6/Beclin1 might be useful ways to monitor autophagy^43^.

It has been recognized that western-style diets, which are characteristic of high levels of n-6 PUFAs, SFAs and trans fatty acids but low levels of n-3 PUFAs, may be associated with an increasing incidence of metabolic syndromes that were induced by inflammatory responses^44^. However, substitution of SFAs with MUFAs would reduce inflammatory responses^6^, and a MUFA–enriched Mediterranean diet can reduce the incidence of metabolic syndromes^13^.

Inflammation could be induced by ROS, which are produced during the process of oxidative phosphorylation. However, ROS are essential for several physiological functions, e.g., maintaining immune function and acting as regulatory mediators in signaling processes^16, 45^. Antioxidant systems play a role in reducing the ROS to a balanced level, and the major antioxidant systems are CAT, SOD and GSH-PX^46^. The fish oil group showed the lowest level of oxidative stress and the highest antioxidant activities in the liver. The soybean oil group showed a great similarity to the fish oil group in the levels of oxidative stress and antioxidant activities. This is because n-3 fatty acids can increase CAT and SOD activities^47^. Supplementation with adequate α-linolenic acid (ALA) or EPA + DHA increased hepatic SOD activity in young rats^48^. It was also reported that EPA or DHA supplementation enhanced the total anti-oxidant status and resistance to lipid peroxidation in young rats^49^. ROS may induce the oxidization of DNA, proteins and lipids during the progression of chronic inflammatory and degenerative diseases^10^. NF-κB is a transcription factor that plays an important role in various inflammatory signaling pathways. It regulates several cytokines, chemokines, adhesion molecules and inducible effector enzymes^50^. The n-3 fatty acids may inhibit NF-κB activation^7^. Glutathione would enhance the n-3 PUFA-induced inhibition to NF-κB activation^51^. SFAs in lard may activate toll-like receptor 4 (*TLR4*) and NF-κB^52^. NF-κB -induced proteins include COX-2, TNF-α, IL-1β, and IL-6, which are in turn potent NF-κB activators, forming an auto-activation loop^53^. Lower NF-κB mRNA level in the soybean oil group was probably due to lower SFAs (16.87%) compared to the lard group (43.59%). A human study confirmed that natural killer cell activity and *in vitro* secretion of IL-1β and TNF–α in young healthy men were significantly reduced by supplementation with 6 g DHA/d for 90 d^54^.

In the present study, dietary fats triggered significant phenotype changes of inflammation in middle-aged rats. The rats fed lard seemed more susceptible to inflammation by counting white blood cells and platelets than those of soybean and fish oils. In fish oil, a relatively high level of palmit oleic acid (16:1) could be involved in the regulation of insulin sensitivity, the inhibition of inflammation and the prevention of fat accumulation in the liver^55^. In addition, ARA can be converted to prostaglandins and leukotrienes that are important pro-inflammatory mediators and can induce ROS production^56^. Linoleic acid in soybean oil can be converted to ARA^57^. Moreover, supplementation with olive oil (high in OA) may cause severe inflammation by up-regulating intrahepatic gene expression of proinflammatory molecules^12^. Higer oleic and linoleic acids in soybean oil might induce greater level of inflammation than fish oil.

The higher level of LDL in the lard group could be associated with inflammation. LDL is rich in cholesterol and cholesteryl ester, which are easily oxidized and aggregated. Oxidized LDL would induce *TLR4*-mediated signaling pathway (*CD36–TLR4–TLR6* inflammasome), and activate NF-κB and Pro-IL-1β that can be cleaved by caspase 1 to IL-1β and further induces inflammation^58^.

PCR array results further confirmed that dietary fats induced different levels of inflammation. As compared to the lard group, intake of soybean oil caused the down-regulation of 16 genes involved in inflammatory response, and 19 inflammation-related genes. The pathway enrichment analysis indicated that intake of soybean and fish oils might reduce inflammatory response by down-regulating gene expression involving complement activation and toll-like receptor signaling. In addition, genes involving oxidative stress and mitochondrial dysfunction were also down-regulated in the fish oil group, and this was in line with the changes of hepatic antioxidant enzyme activities. The intakes of soybean and fish oils resulted in the down-regulation of *FOXO 1*, which might be due to less hepatic fat accumulation, and thus decrease the insulin level. However, higher insulin may activate NF-κB by the phosphorylation of *FOXO* via the *PI3K/Akt* signaling pathway, and result in the down-regulation of MnSOD and CAT. This would increase ROS level, which may in turn activate NF-κB, and as a result, increase inflammation^53^.

Oxidative-stress-induced inflammation is commonly accompanied with telomere dysfunction^17^. Telomeres are long hexamer (TTAGGG) repeats that protect the genome against chromosomal instability and cellular senescence^59^. Exogenous and endogenous ROS can cause telomere attrition and senescence^60^. Senescence would compromise tissue repair and regeneration, and further induce tissue and organism to aging. Telomere length (TL) is considered a potential biomarker of aging^61^. In the present study, the fish oil group had higher aTL than the lard group, indicating that fish oil may prevent DNA damage. This could be attributed to the roles of n-3 PUFAs^59^. Docosapentaenoic acid (DPA) and EPA were also shown to exert a protective effect in old rats by significantly decreasing age-related microglial activation and 8-OHdG (marker of DNA oxidative damage) compared to the young^62^. During aging, inflammation and ROS may activate caspase and further induce cellular apoptosis and senescence^63^. Caspase 1 (*CASP 1*) and clusterin (*CLU*) can activate apoptosis. Lower *CASP 1* and *CLU* mRNA levels were observed in the fish oil and soybean oil groups, which is indicative of less senescence. Age-related ROS generation can stimulate NF-κB activation, and ROS production may be in turn activated by cellular senescence^53^. The present study also showed that the genes related to DNA binding, apoptosis, proteostasis, laminopathies and neurodegeneration were down-regulated by intake of fish oil. In this regard, the substitution of lard for fish oil may retard the aging process at middle ages.

It is noting that effect of dietary fats on inflammation at normal dose was relatively small. However, the diet effect may be enhanced if diet fat dose increases. Even so, the present study still provided significant results of some physiological responses. To a certain extent, the results reflected gradual oxidative stress and inflammation induced by diet fats at middle ages. Further work should be done to compare diet fat effects among young, middle and old ages, and to evaluate the diet effect at higher doses, In addition, autophagy-related markers should be quantified to evaluate whether different source of fats induce autophagy at middle ages.

**Figure 6:**
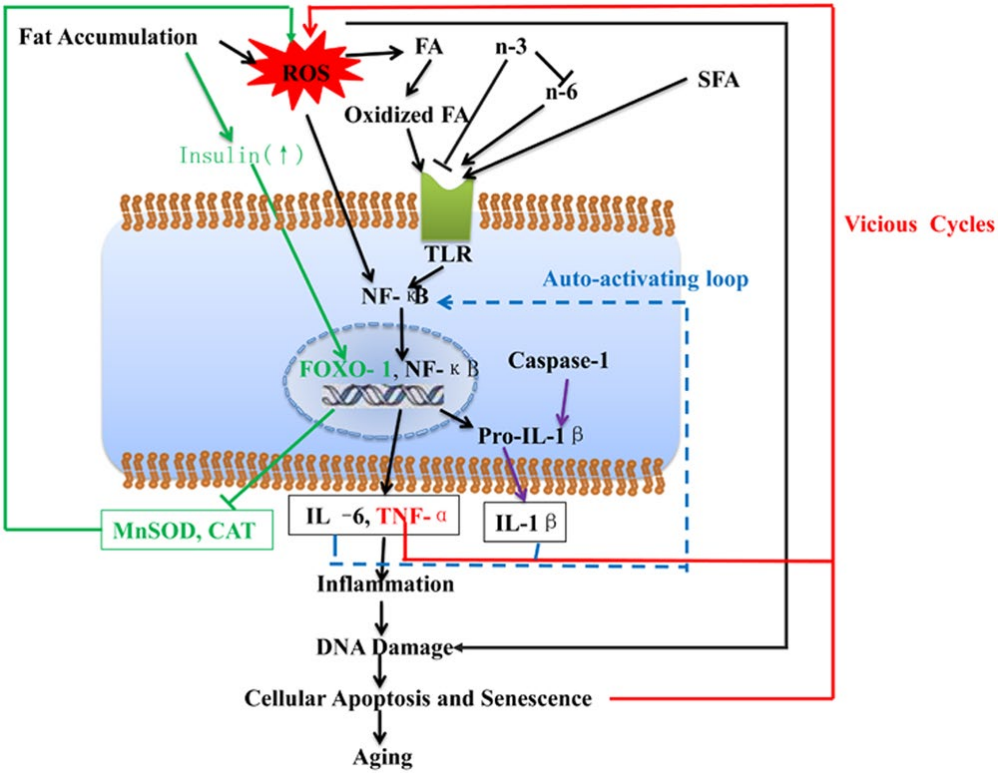
A proposed underlying mechanism for fat-induced oxidation/inflammation/aging. ROS, reactive oxygen species; FA: fatty acids; n-3: n-3 fatty acids; n-6: n-6 fatty acids; SFA: saturated fatty acid; *TLR*: toll- like receptor; NF-κB: nuclear transcription factor κB; *FOXO* 1: fork head box O 1; Pro-IL-1β: pro-interleukin 1β; MnSOD: Mn superoxide dismutase; CAT: catalase; IL-6: interleukin 6; TNF-α: tumor necrosis factor α; IL-1β: interleukin 1β; purple line indicates the *CASP-1* mediated pathway; green line indicates the insulin-induced pathway; red line indicates TNF-α, cellular apoptosis and senescence pathways which increase ROS production; blue dot line indicates auto-activation loop induced by IL-6 and IL-1β.

In summary, an underlying mechanism for the fat-induced oxidation-inflammation in middle-aged rats was proposed (Fig. 6). Fat accumulation may increase ROS production, which can induce the oxidation of proteins, lipids, and DNA, and consequently the cellular apoptosis, senescence and aging. SFAs, oxidized fatty acids and n-6 PUFAs in diets could be transported into the cytoplasm by *TLRs*, and then activate NF-κB and induce inflammation. However, n-3 PUFAs could suppress *TLR*-induced NF-κB activation. ROS may directly activate NF-κB, and induce *CASP-1* to cleaving pro-IL-1β to produce IL-1β. TNF-α, IL-6 and IL-1β could in turn activate NF-κB and further aggravate pro-inflammation. TNF-α, apoptosis and senescence would induce the ROS production. In addition, insulin could activate *FOXO 1*, but inhibit MnSOD and CAT, and as a consequence enhancing ROS production.

## Materials and Methods

### Ethics statements and animals

Animal experimental protocols were reviewed and approved by the Ethical Committee of Experimental Animal Center of Nanjing Agricultural University. All experiments were performed in accordance with the relevant guidelines and regulations of the Ethical Committee of Experimental Animal Center of Nanjing Agricultural University, and all efforts were made to minimize animal pains. Thirty-three male *Sprague Dawley* rats (800.4 ± 8.08 g, 12 months of age) were purchased from the Academy of Military Medical Science Laboratory Animal Center (Beijing, P.R. China, SCXK < Jun > 2012-0004). All the rats were housed individually in plastic cages in a specific pathogen-free animal center (SYXK < Jiangsu > 2011-0036). The room was kept at a temperature of 20 ± 0.5 °C and humidity of 60 ± 10% on a 12 h light–dark schedule. All the rats were given free access to diets and water.

### Diets

The diets were formulated according to the AIN-93M protocol and prepared by Trophic Animal Feed High-Tech Co., Ltd (Nantong, China). The diets differed in the nature of the fats, which were soybean oil, lard and fish oil. Soybean oil was purchased from Shanghai Jiali Oil Industry Co. Ltd (Shanghai, China), and lard was obtained from Tianjin Lihongde Fat Products Inc. (Tianjin, China). Fish oil was obtained from Rongcheng Ayers Ocean Bio-technology Co, Ltd (Weihai, China). The fatty acid profiles of three fats are shown in Table S3. The diets were formulated as (per 1000 g diet): 465.69 g cornstarch, 140.0 g casein, 155.0 g dextrinized cornstarch, 100.0 g sucrose, 40.0 g soybean oil, lard or fish oil, 50.0 g fiber, 1.8g L-cystine, 2.5 g choline bitartrate, 10.0 g vitamin mixture, 35.0 g mineral mixture and 0.014 g tertbutylhydroquinone. The diets were vacuum packaged, stored at –20 °C and allowed to reach room temperature before being served.

### Animal feeding and sampling

The rats were randomly assigned to one of the three diet groups, i.e. soybean oil, lard and fish oil (n = 11, each). Feed intake was recorded daily and body weights were recorded weekly. After 90-day feeding, all rats were decapitated. Blood was collected into EDTA-Na_2_ treated tubes and plasma was centrifuged at 1750 × g for 10 min. The liver was weighed and the largest hepatic lobe was cut into 5 10 × 10 mm cubes at the same part. One cube was fixed in 4% paraformaldehyde for Oil Red O staining, and the other cubes were snap-frozen and stored at −80 °C until analysis.

### Hepatic fat accumulation analysis

After 12 h fixation in 4% paraformaldehyde at 4 °C, the liver samples were embedded in paraffin and 10 μm - thick sections were cut. The sections were stained with Oil Red O according to the procedures of Goto-Inoue *et al*.^64^. The sections were examined under a light microscope with a magnification × 200. Triacylglycerol and total cholesterol were analyzed with commercial kits according to the manufacturer’s protocols (Nanjing Jiancheng Bioengineering Institute, Nanjing, China).

### Blood profiling analysis

Plasma parameters were measured under an automatic biochemical analyzer (DXC − 800, Beckman Coulter Inc., Fullerton, CA, USA) and an automatic hematology analyzer (SYSMEXLAS, Sysmex Corporation, Kobe, Japan). Insulin was determined with a radioimmunoassay kit according to the manufacturer’s instruction (Beijing North Institute of Biological Technology Company, Beijing, China). IRI was calculated as follows^65^:

1 $${\rm{IRI}}=(\mathrm{fasting}\,{\rm{insulin}}\,{\rm{in}}\,\mathrm{mU}/{\rm{L}}\times \mathrm{fasting}\,{\rm{glucose}}\,{\rm{in}}\,\mathrm{mM})/\mathrm{22.5.}$$

### Hepatic oxidative stress analysis

Liver samples (0.5 g) were homogenized in 4.5 mL ice-cold physiological saline for 1 min and then centrifuged (2700 × g, 4 °C, 10 min). The supernatants were aliquoted and the enzymatic activities of CAT, SOD, GSH-PX, and T-AOC were analyzed with commercial kits (Nanjing Jiancheng Bioengineering Institute, Nanjing, China) according to the manufacturer’s protocols.

### RNA isolation and RT- PCR analysis of inflammatory cytokines

Total RNA was extracted from liver samples using RNeasyPlus Mini Kit (Qiagen, Hilden, Germany) according to the manufacturer’s instructions. The purity and quantity of total RNA were measured by a NanoDrop 2000 spectrophotometer (Thermo Fisher Scientific, Waltham, MA, USA) at 260 and 280 nm. The integrity of RNA samples was evaluated by agarose gel electrophoresis. First-strand cDNA was synthesized from 250 ng of total RNA using RT^2^ First Stand kit (Qiagen, Hilden, Germany) in a Veriti® Certified Refurbished Thermal Cycler (Thermo Scientific, USA) according to the manufacturer’s instructions. The RT-PCR reactions were run using SYBR Premix Ex Taq Kit (Takara Biotechnology Co. Ltd., China) in QuantStudio™ 6 Flex Real-Time PCR System (Thermo Scientific, USA). Primers were designed according to the public database at the National Center for Biotechnology Information (NCBI) (http://www.ncbi.nlm.nih.gov/RefSeq/) and were synthesized by Sangon Biotech Co., Ltd (Sangon, Shanghai, China). Primers used for RT-PCR were presented in Supplementary Table S4. The amplification was performed in a total volume of 20 μL, containing 10 μL of SYBR Premix Ex Taq, 0.4 μL of each primer (10 μM), 0.4 μL of ROX Reference Dye II (Takara Biotechnology Co. Ltd., China), 2 μL of cDNA and 6.8 μL of sterilized doubled-distilled water. The RT-PCR program was as follows: 95 °C for 10 min, 35 cycles of 95 °C for 30 s, 60 °C for 30 s and 72 °C for 30 s, holding at 72 °C for 10 min, and the fluorescence signals were collected at 60 °C. Each sample was performed in triplicate. Relative mRNA levels were calculated using the 2^−ΔΔCt^ method. β-actin was applied as reference gene to determine NF-κB, IL-1β and TNF-α levels, and GADPH was applied to determine IL-6 level.

### Aging - related RT-PCR array analysis

After RNA isolation of liver and first-strand cDNA synthesis (see subsection “RNA isolation and RT-PCR analysis of inflammatory cytokines”), a RT² Profiler™ PCR array rat aging kit (330231 PARN-178ZA, Qiagen, Hilden, Germany) and a RT^2^ SYBR Green ROX qPCR kit (330522, Qiagen, Hilden, Germany) were applied to evaluate aging-related gene expression on an ABI 7500 Real-Time PCR System (Applied Biosystems, Foster City, CA, USA) according to the manufacturer’ protocols. All RNA samples were normalized to 250 ngbefore first-strand cDNA synthesis. The RT^2^ Profiler PCR array profiled the expression of 84 target genes besides housekeeping and control genes (Supplementary File S1). The RT-PCR cycling conditions were as follows: 95 °C for 10 min, 40 cycles of 95 °C for 15 s, 60 °C for 1 min, 95 °C for 1 min, and 65 °C for 2 min to collect fluorescence signals. Differentially expressed genes were obtained by using an online program (Geneglobe; Qiagen, Redwood City, CA; http://pcrdataanalysis.sabiosciences.com/pcr/arrayanalysis.php) in which the lard group was set as control, and *RPLP1* was set as housekeeping gene for normalization. The genes with greater than 1.2-fold changes were defined as differentially expressed genes. The protein-protein interactions of significant gene products were evaluated by the STRING 10 (http://string-db.org/) and visualized by the Cytoscape 3.3.0.

### Telomere length

Absolute telomere length (aTL) was measured with a RT-PCR method according to O’Callaghan and Fenech^66^ with minor modifications. Briefly, DNA was isolated with a QIAamp DNA mini kit (Qiagen, Hilden, Germany) according to the manufacturer’s protocols. The RT-PCR reactions were run in QuantStudio™ 6 Flex Real-Time RCR System (Thermo Scientific, Carlsbad, USA) using a SYBR Premix Ex Taq Kit (Takara Biotechnology Co. Ltd., China). The cycling profile consists of denaturation at 95 °C for 10 min, denaturation at 95 °C for 15 s, annealing at 60 °C for 60 s, extension at 72 °C for 30 s, holding at 72 °C for 10 min with data collection, and 40 cycles with fluorescence data collection. Standard oligomers and primers were shown in Supplementary Table S5. The aTL was calculated as follows:

2 $$\mathrm{aTL}=\mathrm{amount\; of\; telomere\; sequence\; per\; reaction}({\rm{kb}})/\mathrm{genome\; copy\; number}$$

### Statistical analysis

The PCR array data was conducted by t-test between two variances with an online program (http://pcrdataanalysis.sabiosciences.com/pcr/arrayanalysis.php, Geneglobe, Qiagen, Redwood City, CA). The other variables were analyzed using the analysis of variance under the SAS program (version 9.2, SAS Institute Inc., Cary, NC, USA). The differences between diet groups were compared by the procedure of Student-Newman-Keuls Test. The values were considered significantly different if the *P* value was less than 0.05.

## Electronic supplementary material

Supplemetary Information 1

Supplementary File S1
