# Supplementary material for: Fish oil diet may reduce inflammatory levels in the liver of middle-aged rats
Source: Sci Rep. 2017 Jul 24;7:6241. doi: 10.1038/s41598-017-06506-3 (PMC5524965; doi:10.1038/s41598-017-06506-3)
Supplement: Supplementary file 1 — Supplemetary Information 1 [file 41598_2017_6506_MOESM1_ESM.pdf]

Supplementary Information 1

Tittle of manuscript: Intake of fish oil, lard and soybean oil induced different inflammatory effects to middle-aged rats

Authors: Yingqiu Li, Fan Zhao; Qiayu Wu; Mengjie Li; Yingying Zhu; Jing Zhu; Shangxin, Song; Yafang Ma; He Li; Xuebin Shi; Xinglian, Xu; Chunbao Li; Guanghong Zhou.

Table S1 Pathway enrichment analysis of differentially expressed genes of soybean oil group

	Wikipathways	Wikipathways code	*P*-value
1	Complement and coagulation cascades	WP449	6.303E-10
2	Oxidative damage	WP1496	3.441E-9
3	Complement activation, classical pathway	WP200	2.692E-7
4	Toll like receptor signaling	WP88	0.006

Table S2 Pathway enrichment analysis of differentially expressed genes of the fish oil group

	Wikipathways	Wikipathways code	*P*-value
1	Complement and coagulation cascades	WP449	6.769E-9
2	Oxidative damage	WP1496	9.531E-7
3	Complement activation, classical pathway	WP200	9.580E-7
4	Toll like receptor signaling	WP88	0.01061
5	Mitochondrial gene expression	WP1263	0.04272
6	FAS pathway and stress induction of HSP regulation	WP571	0.01244

Table S3 Lipid profile of dietary fats (g / 100g oil)

Items	Soybean oil	Lard	Fish oil
C10:0	0	0.05	0
C12:0	0	0.06	0
C14:0	0.06	1.17	1.13
C15:0	0	0	0.41
C16:0	10.65	24.79	20.64
C16:1	0.07	1.25	5.51
C17:0	0.05	0.25	1.15
C17:1	0.03	0.13	0.36
C18:0	4.86	16.11	6.72
C18:1n9t	0	0.21	0.19
C18:1n9c	23.44	37.60	17.59
C18:2n6c	53.07	15.65	2.48
C20:0	0.20	0.21	1.26
C18:3n6	0.60	0	0.23
C20:1	0.17	0.81	2.97
C18:3n3	5.75	0	1.85
C21:0	0.61	0.74	0.07
C20:2n6	0	0.61	0.25
C22:0	0.34	0	4.03
C20:3n6	0	0.08	2.61
C21:3n9	0	0	0.13
C20:4n6	0	0.07	0.11
C23:0	0	0.19	1.37
C22:2n6	0	0	0.75
C24:0	0.09	0	0
C20:5n3	0	0	15.79
C24:1	0	0	0.12
C22:6n3	0	0	12.28
SFA	16.87	43.59	36.78
MUFA	23.71	39.99	26.74
PUFA	59.43	16.41	36.48
PUFA/SFA	3.52	0.38	0.99
n6	53.67	16.41	6.43
n3	5.76	0	29.91
n3/n6	0.11	0	4.65

Table S4 RT-PCR primers for related genes and β-actin

Gene	Primer sequence (5' to 3')	Product length (bp)	GenBank accession No.
NF-κB	F: CACGGATGACAGAGGCGTGTATAAGG<br>R: GGCGGATGATCTCCTTCTCTCTGTCTG	130	XM_006233360.2
IL-1β	F: TCTGTGACTCGTGGGA<br>R: GTCTGTGCTCTGCTTGA	432	NM_031512.2
TNF-α	F: CATGATCCGAGATGTGGAACTGGC<br>R: CTGGCTCAGCCACTCCAGC	316	XM_008772775.1
IL-6	F: AAGGACCAAGACCATCCAAC<br>R:ACCACAGTGAGGAATGTCCA	129	NM_012589.2
β-actin	F: CAGGATGGCGTGAGGGAGAGC<br>R: AAGGTGTGATGGTGGGAATGG	407	NM_031144.3
GADPH	F: TGACAACTCCCTCAAGATTGTCA<br>R: GGCATGGACTGTGGTCATGA	121	NM_017008.4

Table S5 Oligomers used for absolute telomere length assay in rat liver

Items	Oligomer Name	Oligomer sequence (5' to 3')	Amplicon size(bp)
Standards	Telomere standard	（TTAGGG）$_{14}$	84
	36B4 standard	ACTGGTCTAGGACCCGAGAAGACCTCCTTCTTCCAGGCT TTGGGCATCACCACGAAAATCTCCAGAGGCACCATTGA	77
PCR Primers	Telo F	CGGTTTGTTTGGGTTTGGGTTTGGGTTTGGGTTTGGGTT	76
	Telo R	GGCTTGCCTTACCCTTACCCTTACCCTTACCCTTACCCT	
	36B4 F	ACTGGTCTAGGACCCGAGAAG	78
	36B4 R	TCAATGGTGCCTCTGGAGATT	

Figure S1 Antioxidant enzyme activities level of rats fed with soybean oil, lard and fish oil diets.

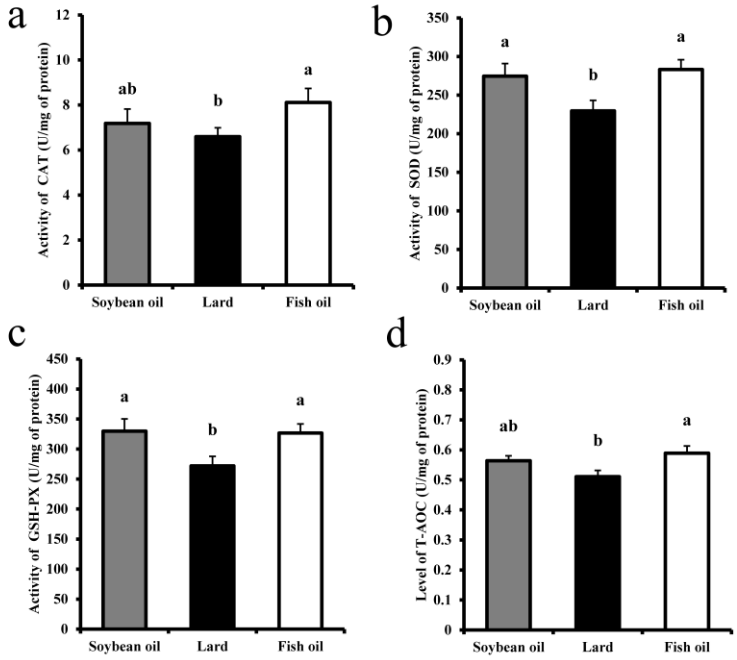

(a) Catalase activity; (b) Superoxide dismutase activity; (c) Glutathione peroxidase activity; (d) Total antioxidant capacity. Values are shown as means ± SE (n =11). Different letters indicate significant difference ($P < 0.05$)
